# Dynamics of T-Cell Intracellular Antigen 1-Dependent Stress Granules in Proteostasis and Welander Distal Myopathy under Oxidative Stress

**DOI:** 10.3390/cells11050884

**Published:** 2022-03-04

**Authors:** Andrea Fernández-Gómez, Beatriz Ramos Velasco, José M. Izquierdo

**Affiliations:** Centro de Biología Molecular Severo Ochoa, Consejo Superior de Investigaciones Científicas, Universidad Autónoma de Madrid (CSIC/UAM), C/Nicolás Cabrera 1, Campus de Cantoblanco, 28049 Madrid, Spain; afernandez@cbm.csic.es (A.F.-G.); bramos@cbm.csic.es (B.R.V.)

**Keywords:** TIA1, oxidative stress, stress granules, Welander distal myopathy

## Abstract

T-cell intracellular antigen 1 (TIA1) is an RNA-binding protein that is primarily involved in the post-transcriptional regulation of cellular RNAs. Furthermore, it is a key component of stress granules (SGs), RNA, and protein aggregates that are formed in response to stressful stimuli to reduce cellular activity as a survival mechanism. TIA1 p.E384K mutation is the genetic cause of Welander distal myopathy (WDM), a late-onset muscular dystrophy whose pathogenesis has been related to modifying SG dynamics. In this study, we present the results obtained by analyzing two specific aspects: (i) SGs properties and dynamics depending on the amino acid at position 384 of TIA1; and (ii) the formation/disassembly time-course of TIA1^WT/WDM^-dependent SGs under oxidative stress. The generation of TIA1 variants—in which the amino acid mutated in WDM and the adjacent ones were replaced by lysines, glutamic acids, or alanines—allowed us to verify that the inclusion of a single lysine is necessary and sufficient to alter SGs dynamics. Moreover, time-lapse microscopy analysis allowed us to establish in vivo the dynamics of TIA1^WT/WDM^-dependent SG formation and disassembly, after the elimination of the oxidizing agent, for 1 and 3 h, respectively. Our observations show distinct dynamics between the formation and disassembly of TIA1^WT/WDM^-dependent SGs. Taken together, this study has allowed us to expand the existing knowledge on the role of TIA1 and the WDM mutation in SG formation.

## 1. Introduction

T-cell intracellular antigen 1 (TIA1) is an RNA-binding protein identified to be associated with cytotoxic granules of T lymphocytes [1]. Structurally, the protein consists of three RNA-binding motifs (RRM) and a carboxyl(C)-terminal domain of low complexity [2]. TIA1 is expressed in a cell- and tissue-dependent manner in the organism [3,4]. It is distributed between the nucleus and the cytoplasm, and plays an important role in the regulation of gene expression and RNA metabolism [5,6]. In the nucleus, the main function of TIA1 is to regulate the splicing of some pre-mRNAs, although it is also involved in the regulation of transcription [7,8,9,10,11,12,13]. In the cytoplasm, however, it acts as a translational repressor, both under conditions of homeostasis and, mainly, stress [5,14,15,16]. In this situation, ribosome assembly and translation are inhibited [14,15,16]. TIA1 can then bind to translation initiation factors and to the small subunit of ribosomes, forming complexes that interact with mRNA and aggregate thanks to the C-terminal prion domain of TIA1, among others. These aggregates, called stress granules (SG), prevent the translation of some mRNAs, and favor the translation of those that give rise to proteins that help to overcome stress and recover homeostasis [14,15,16]. Other processes in which TIA1 participates are transport, subcellular localization, and mRNA stability [5,6,17,18].

The p.E384K mutation of TIA1 is the genetic cause of Welander distal myopathy (WDM). It is an autosomal dominantly inherited muscular dystrophy, which manifests late (40–60 years) in populations in Sweden and some regions of Finland [19,20]. It affects the muscles of the hands and lower extremities [21,22]. All individuals with MDW share a rare haplotype on chromosome 2p13 where a heterozygous missense founder mutation (c.1150G > A; p.E384K) was identified in the TIA1 gene, in the region encoding the C-terminal domain [23,24]. In the first study in which it was identified, the mutation was associated with alterations in the alternative processing/splicing of the pre-mRNA of the SMN2 (survival motor neuron 2) gene, and in the second, with the dynamics of SG formation [23,24]. It has recently been confirmed in a cellular model of the disease that the expression of the mutated version of the protein mainly affects the dynamics of SGs, apoptosis, and mitochondrial dynamics [25].

SGs are non-membranous aggregates that are formed from mRNA arrested at translation initiation, and thus contain eIFs, in addition to both RNA-binding proteins and proteins with other functions [14,15,16,26,27,28]. Like other non-membranous intracellular compartments, such as processing bodies or Cajal bodies, SGs are formed by a process called phase separation. In the case of SGs, this phase separation is liquid–liquid (LLPS) [26,27,28,29]. This implies that, within the cell, these regions change their material properties, which come to resemble those of liquids or gels, and become differentiated from the rest of the cytoplasm without the need to be separated by a membrane [26,27,28]. Interestingly, upon certain endogenous (starvation, unfolded protein response, oxidative stress, DNA damage, etc.) or exogenous (heat, cold, hyperosmotic, or chemical shock) stress situations, the cell can trigger the LLPS of specific proteins, and allow only other specific proteins to also partition into the formed condensates, so that their composition remains specific [28,29,30]. Proteins with low-complexity and intrinsically disorganized prion-like domains (PLDs), as is the case of the C-terminal domain of TIA1, are known to have the ability to target these LLPS. In fact, it has been shown that TIA1 is able to induce it not only in vivo, associated with RNA and other proteins to form SGs [15,26,31], but also in vitro, self-aggregating in the presence of RNA or single stranded DNA for which it has high affinity [30,32]. Although these C-terminal domains of TIA1 are known to interact with each other during SG formation and remain disorganized, the exact arrangement of the protein within the aggregates is unknown, although a model has been proposed whereby TIA1 gives rise to micellar-like structures [29].

Despite the fact that the sequence-intrinsic features that drive LLPS or control the material properties of the resulting aggregates are not yet fully understood; recently, the existence of a “molecular grammar” according to which interactions between specific amino acids can either promote or impede the process has been demonstrated [28,29]. These studies show that the interaction between RRM domains and PLDs is necessary to trigger LLPS. For example, in the fused in sarcoma (FUS) family of proteins, it has been observed that the interactions between the tyrosine (aromatic) residues of the PLD and the arginine (positively charged) residues of the RRMs determine the saturation concentration at which LLPS takes place. However, electrostatic interactions between these two types of domains, favored by charged amino acids that attract or repel each other, are also an important factor in the process. Finally, some PLD amino acids, such as glycine, favor aggregates to maintain a liquid consistency, while others such as glutamine trigger their hardening [28]. Taken together, studies show that amino acid properties—such as polarity, aromaticity, charge, and size—strongly influence their ability to interact with other protein regions and trigger LLPS that results in the formation of SGs [28,29].

In recent years, the study of SGs as structures with an important role in the development and progression of different diseases has gained momentum, following the detection of mutations in proteins, including TIA1, that increase the formation and persistence of SGs [33,34]. In other cases, are other altered proteins that form pathological aggregates in which TIA1 is also found. These diseases in which dysfunctional SGs containing TIA1 are generated are mainly of the nervous system. Thus, it has been seen that some mutations in the TIA1 gene are associated with the pathology of amyotrophic lateral sclerosis [35,36], and that it also forms aggregates in tauopathies, such as Alzheimer’s disease [30,37], and in Huntington’s disease [38]. However, dysfunction of SGs due to TIA1 mutations has also been linked to muscle diseases, most notably Welander distal myopathy (WDM) [23,24].

While its genetic cause is known, WDM has been poorly studied. However, understanding the molecular background of the disease—i.e., the alterations in cellular homeostasis derived from the E384K mutation of TIA1—is essential in order to find therapeutic opportunities, which are so far nonexistent. That is why the main objective of this work has been to continue to deepen our understanding of the molecular and cellular details associated with the expression of the mutated version of TIA1 during the development of WDM. Specifically, we have sought to exacerbate the abnormalities observed in the dynamics of formation, assembly, and disassembly of SGs dependent on the mutated version of TIA1 under conditions of stress or homeostasis. To this end, new variants have been generated to replace the original glutamic acid present in the wild-type (WT) version of TIA1 with three lysines (K), three glutamic acids (E), or three alanines (A). The results suggest that the inclusion of a single lysine is necessary and sufficient to alter TIA1-dependent SGs dynamics.

## 2. Materials and Methods

### 2.1. Cell Culture

HEK293 Flp-In T-REx cells (human kidney embryonic cells; from now on HEK-Flp; Invitrogen) were cultured as previously described [25,39,40]. The modified lines FT293 GFP-TIA1a^WT^ and FT293 GFP-TIA1a^WDM^, which express these fusion proteins in a tetracycline-inducible manner, had been previously generated using the Flp-In T-REx system and were cultured as described [25]. All the lines were regularly diluted upon reaching ≃90% confluence and were cultured in an incubator (Thermo Electron Corporation) at 37 °C, with 95% humidity and 5% CO_2_.

### 2.2. Generation of Mutated Versions of TIA1a by Site-Directed Mutagenesis

Plasmids pcDNA-5/FRT/TO-GFP-TIA1a^WT^ (glutamic acid at position 384 of TIA1) and pcDNA-5/FRT/TO-GFP-TIA1a^WDM^ (lysine at position 384) had been previously generated [25]. The other three plasmids for the expression of the modified protein were generated by a “Round the horn PCR” directed mutagenesis procedure [41] using the plasmid pcDNA-5/FRT/TO-GFP-TIA1a^WT^ as a template. This strategy was used to modify in the nucleotide sequence of TIA1 the codons that code for the amino acid that changes in the WDM and those immediately adjacent (modifications p.YET383-385KKK, p.YET38-EEE, and p.YET383-385AAA). It is an adaptation of the inverted polymerase chain reaction (PCR) in which two primers are designed that pair with the template plasmid at the point where the sequence needs to be modified, one with each chain and in opposite directions. One of them also carries the modification. In this case it was a nine-nucleotide modification, and the desired sequence was introduced at the 5′ end of the Forward primer. The 3 Forward primers (F1-3xLys, F2-3xGlu, F3-3xAla) and the Reverse one that were designed as followed: F1-3xLys. sense: 5′-AAAAAAAAACAGTGAGGATCCACTAGTCCAGTG-3′; F2-3xGlu.sense: 5′-GAAGAAGAACAGTGAGGATCCACTAGTCCAGTG-3′; F3-3xAla.sense: 5′-GCAGCAGCACAGTGAGGATCCACTAGTCCAGTG-3′; oligo reverse: 5′-CCCTGCCACTCGATACCCAGAAG-3′. The corresponding PCRs were carried out under the following cycle conditions: 94 °C 5 min, 30 cycles of 94 °C 1 min, 60 °C 1 min and 72 °C 9 min, ending with 72 °C 10 min. The products were digested with DpnI (BioLabs, San Diego, CA, USA) for 1 h at 37 °C to eliminate the template plasmid, after which they were ligated with T4 DNA ligase (BioLabs, San Diego, CA, USA) for 1 h at RT, following the manufacturer’s instructions. The resulting constructs were transformed into *Escherichia coli* DH5α by heat shock (42 °C, 1.5 min). The sequence of all the constructions was verified by automatic DNA sequencing (Macrogen Spain) using BGH primer (5′-GGCAACTAGAAGGCACAG-3′).

### 2.3. Cell Transfection and Oxidative Stress Induction

HEK-Flp cells were transfected, at 50% confluence, with 2 μg of the corresponding plasmid (pcDNA-5/FRT/TO-GFP-TIA1a^WT^, -GFP-TIA1a^WDM^, -GFP-TIA1a^KKK^, -GFP-TIA1a^EEE^, and -GFP-TIA1a^AAA^) using TurboFect (Thermo Scientific, Waltham, MA, USA) according to the manufacturer’s instructions. After 2–4 h, protein expression was induced by adding tetracycline (100 ng/mL; Merck, Darmstadt, Germany). To induce oxidative stress, sodium arsenite (0.5 mM; Merck, Darmstadt, Germany) was added 24 h after tetracycline, and cells were collected after 1 h of incubation for analysis. In experiments with recovery, arsenite was removed after 1 h, cells were washed with phosphate-buffered saline (PBS), and fresh culture medium was added. Analysis was performed 3 h later. The induction of FT293 GFP-TIA1a^WT^ and FT293 GFP-TIA1a^WDM^ cells with tetracycline being carried out at ≃ 50% confluence. Oxidative stress was induced as above, and cycloheximide (CHX; 5 μg/mL; Merck, Darmstadt, Germany) was also used, as a control. Recovery was carried out for 3 and 6 h after to removing sodium arsenite.

### 2.4. Protein Purification, Western Blot Analysis, and Staining with Coomassie Blue

Cells were cultured and processed for protein isolation and Western blot analysis as previously described [25,39,40] using the following antibodies: anti-TIA1 (sc-1751 (1/3000), Santa Cruz Biotechnology, Dallas, TX, USA), anti-eIF2α (sc-133132 (1/4000), Santa Cruz Biotechnology, Dallas, TX, USA), anti-eIF2α-P (Ser51) (9721L (1/4000), Cell Signalling Technologies, Danvers, MA, USA), anti-HuR (3A2) (sc-5261 (1/4000), Santa Cruz Biotechnology, Dallas, TX, USA), and anti-tubulin-α (T5168, (1/5000), Merck, Darmstadt, Germany). Secondary antibodies were from goat, rabbit, and mouse (Promega, Madison, WI, USA). Coomassie staining of the gels was performed using Coomassie Brilliant Blue G-250 reagent (Bio-Rad, Hercules, CA, USA).

### 2.5. Microscopy Analysis

For confocal microscopy analysis, HEK293-Flp cells were cultured on coverslips and fixed with 10% formalin (Merck, Darmstadt, Germany). To-Pro3 (1 μM; Invitrogen, Waltham, MA, USA) was used for selective staining of the nucleus. For live cell imaging, FT293 GFP-TIA1a^WT^ and FT293 GFP-TIA1a^WDM^ cells were grown on glass-bottom p35 plates, in complete DMEM medium without phenol red. During these experiments, the incubator of the confocal microscope was kept at 37 °C and 5% CO_2_. ImageJ software was used for image processing. The images were visualized and taken using a confocal microscopy Nikon A1R+ in vivo.

### 2.6. Statistical Analysis

The data are represented as means ± standard errors of the means (SEM). Student’s *t*-test (paired and two-tailed) was applied to determine statistical significance between 2 groups. *p*-values of < 0.05 were considered statistically significant.

### 2.7. Other Software

For in silico prediction of the ability of designed proteins to trigger LLPS, the PSAP classifier algorithm, developed by van Mierlo et al. (2021), was used [42]. The classifier was trained using a set of proteins, provided by van Mierlo et al. (2021), which have been proven to trigger LLPS. Thus, we obtained the default model that was subsequently used to test our protein variants and obtain their PSAP score, or probability to trigger LLPS (https://github.com/vanheeringen-lab/psap (accessed on 22 February 2022)).

## 3. Results

### 3.1. Analysis of the Formation of TIA1-Dependent Stress Granules: Significance of Lysine Residues

To further study the point mutation associated with WDM and its role in the dynamics of SGs, variants of the protein TIA1 were designed in which this amino acid and the two adjacent ones were replaced by triplets of lysines, glutamic acids, and alanines (TIA1a^KKK^, TIA1a^EEE^, and TIA1a^AAA^, respectively) in order to try to enhance the effect of this mutation.

**Figure 1:**
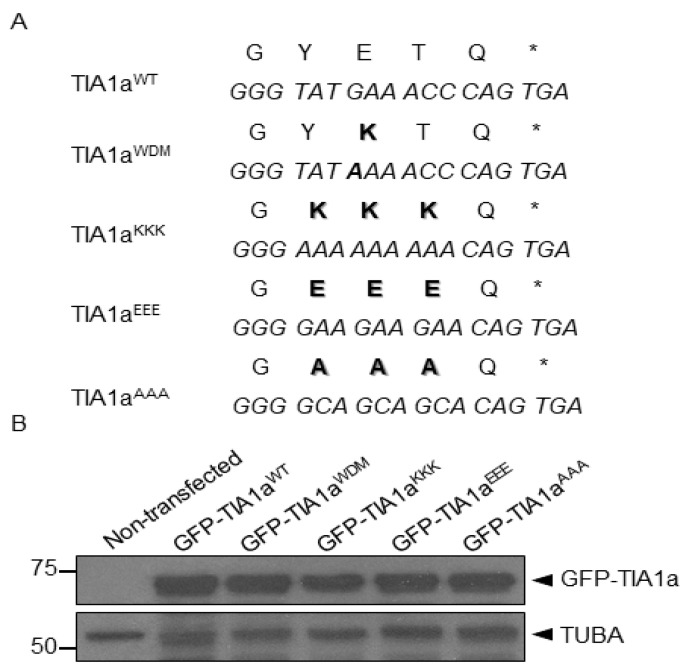
SGs associated with TIA1 expression are dependent on lysine residues. (**A**) pcDNA 5/FRT/TO-GFP-TIA1a plasmids were amplified by PCR around the horn to incorporate either three lysines (KKK), three glutamic acids (EEE), or three alanines (AAA) into TIA1 C-terminal region. The nucleotide triplets of the human TIA1 gene are shown in italic capital letters and the primary amino acid sequence in normal capital letters and boldface. Bold letters highlight amino acid residues changed in plasmid constructs generated by targeted mutagenesis. Asterisks point to the nonsense TGA triplet. (**B**) Western blot of protein extracts from non-transfected HEK293-Flp cells and HEK-Flp cells transfected with GFP-TIA1a^WT^, GFP-TIA1a^WDM^, GFP-TIA1a^KKK^, GFP-TIA1a^EEE^, or GFP-TIA1a^AAA^ using anti-TIA1 and anti- α-tubulin (TUBA) antibodies. Molecular weight (kDa) markers are shown and each band is identified.

The PCR around the horn technique allowed the generation of pcDNA-5/FRT/TO plasmids including the GFP-TIA1a sequence with the three desired modifications (Figure 1). The HEK-Flp cell line was used to analyze the effect of the expression of the variants generated, including the GFP-TIA1a^WT^ and GFP-TIA1a^WDM^ constructs, which we already had. For this purpose, this line was transiently transfected with the five plasmids, and Western blot analysis allowed us to verify that all fusion proteins were expressed correctly and to the same degree, according to the sample loading control associated with endogenous α-tubulin expression (Figure 1).

**Figure 2:**
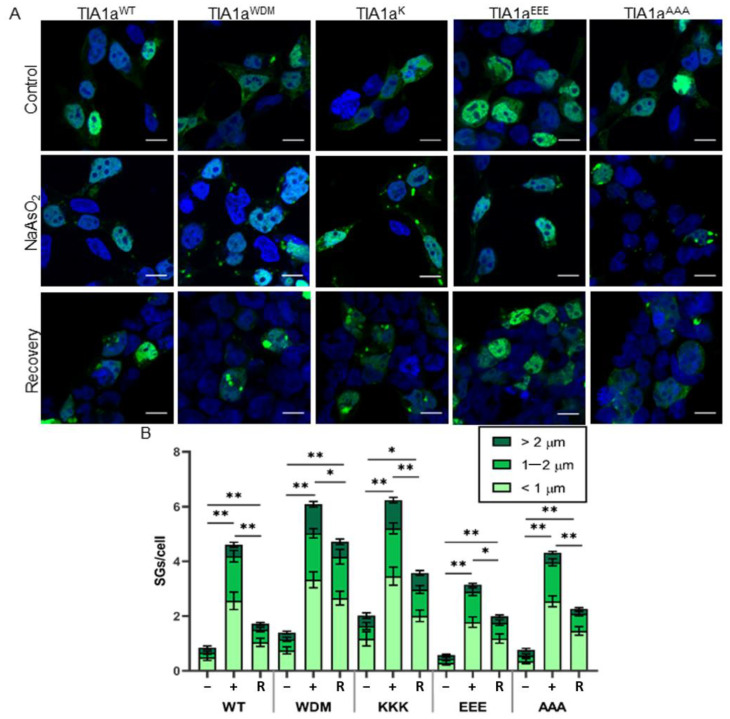
SGs associated with TIA1 expression are dependent on lysine residues. (**A**) Fluorescence microscopy images of HEK-Flp cells transfected with the plasmids indicated in Figure 1 showing expression of TIA1 variants fused to GFP (green). Untreated cells (control), and those treated with sodium arsenite for 1 h and after 3 h recovery after removal of stress are shown. Nuclei were stained with To-Pro3 (blue). The indicated scale corresponds to 10 μm. (**B**) Quantification of the number and size of SGs in the experimental situations described in (**A**). Data represent the mean ± standard error of the mean (*n* = 47–157 cells for each condition from three independent experiments). Significant differences according to Student’s *t*-test are represented for the total number of SGs (* *p* < 0.01; ** *p* < 0.001).

It has previously been shown that expression of the mutated form of TIA1 present in WDM alters both the size and abundance as well as the assembly and disassembly dynamics of SGs [23,24,25]. Therefore, these same parameters were analyzed by confocal microscopy in HEK-Flp cells transfected with the plasmid constructs. We examined the formation of spontaneous and oxidative stress-induced SGs (treatment with 0.5 mM sodium arsenite for 1 h), as well as their evolution 3 h after removal of the stressor (Figure 2A).

First, the results confirmed that the subcellular localization of all GFP-TIA1a variants was as expected for the protein, nucleocytoplasmic with nucleolar exclusion [5,25] (Figure 2A). The differences observed in GFP signal intensity between cells and thus variability in fusion protein expression is the result of transfection efficiency (≃20–30%) and cell culture dynamics. Nevertheless, with all variants, SG formation could be observed in transfected cells, both spontaneously and induced by oxidative stress, which did not completely disappear after 3 h of recovery without the oxidizing agent (Figure 2A).

**Figure 3:**
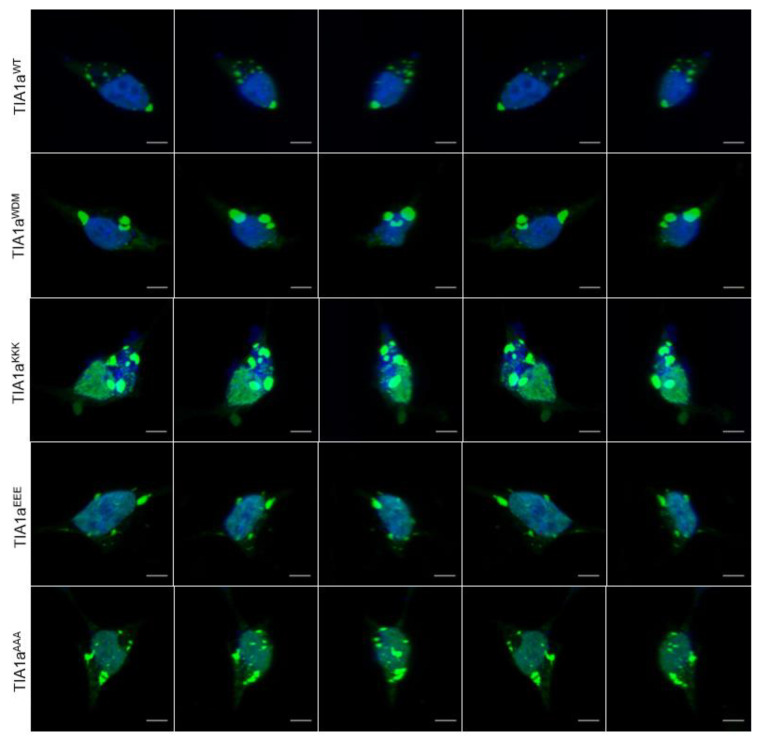
Details of the 3D reconstitution of GFP-tagged TIA1 variants in stress granules under oxidative stress. Fluorescence microscopy images of HEK-Flp cells transfected for 24 h with the plasmids indicated in Figure 1 showing expression of indicated TIA1 variants fused to GFP (green) into stress granules (0.5 mM sodium arsenite for 60 min). Nuclei were stained with To-Pro3 (blue). Scale bars represent 5 μm.

Subsequently, we also performed a count of the total number of SGs per cell in each situation described, as well as their sizes, which were classified into three categories: smaller than 1 μm, between 1 and 2 μm, and larger than 2 μm (Figure 2B). The analysis showed that TIA1a^WDM^ and TIA1a^KKK^ have a moderate but significant ability to generate SGs spontaneously with respect to TIA1a^WT^, which did not exhibit TIA1a^EEE^ or TIA1a^AAA^. These SGs formed in the absence of stress were mostly small in size. Induction with sodium arsenite triggered massive SG formation in cells transfected with all variants. However, TIA1a^WDM^ and TIA1a^KKK^ gave rise to a greater number of SGs than TIA1a^WT^ in the presence of the stressor, and of larger size, as it was the number of SGs larger than 2 μm that showed a significant difference from TIA1a^WT^. However, the analyses showed no difference between the amount of SGs generated by TIA1a^WDM^ and TIA1a^KKK^, so the increase in the number of consecutive lysines around residue 384 of the protein did not intensify the effect already shown by the mutation. At the opposite extreme, TIA1a^EEE^ resulted in fewer SGs than TIA1a^WT^, whereas there was no difference with respect to TIA1a^AAA^. Thus, increasing the number of negatively charged residues in this region of the C-terminal end does negatively affect the ability of the protein to form SGs, but eliminating the E residue in the WT form has no appreciable effect. With these two variants, the proportion of large SGs in the cells was much lower than in TIA1a^WDM^ and TIA1a^KKK^ (Figure 3 and Video S1). Finally, recovery time in the absence of arsenite significantly decreased the number of SGs in all cases, but without reaching basal levels. The smallest decrease occurred in TIA1a^WDM^ and TIA1a^KKK^, although, interestingly, it was greater in the latter case, although the difference was not significant. The behavior of TIA1a^EEE^ and TIA1a^AAA^ was similar to that of TIA1a^WT^, although in proportion to the number of SGs formed, the decreases were less pronounced. It is particularly striking in the case of TIA1a^EEE^, where a lower appearance of SGs was observed upon induction of oxidative stress, but also a delay in their disassembly.

Taken together, the observations indicate that expression of TIA1a^WDM^ = TIA1a^KKK^ > TIA1a^AAA^ > TIA1a^EEE^ leads to an increase in the total number, average size, and stability of SGs under conditions of oxidative stress and subsequent recovery (Figure 3). Therefore, these results suggest that the charge contributed by the amino acids at the C-terminal end of TIA1 influences the ability of TIA1 to interact, aggregate, and trigger LLPS, affecting the fluidity and dynamics of the generated SGs.

On the other hand, van Mierlo et al. have developed an algorithm called PSAP classifier, an in silico approach to determine the probability of proteins to trigger LLPS events [42]. We modified the tool in order to test it with the sequence of TIA1a^WDM^ and the generated variants, and to confirm in silico what we had observed in vivo with HEK-Flp cells. However, the results were not as expected, as it gave all of them low probabilities of generating condensates (TIA1a^WDM^, 0.004; TIA1a^KKK^, 0.008; TIA1a^EEE^, 0.008; TIA1a^AAA^, 0.005; TIA1^WT^ was used to train the algorithm, but then could not be tested), when TIA1 is known to be one of the key proteins that give rise to SGs, and TIA1^WDM^ further potentiates this effect [23,24,25,31]. Our results confirm this assumption, as well as that the generated variants maintain this ability to form SGs. Moreover, the algorithm gave very similar scores to all variants, when we have verified in vivo that there are differences between them in terms of ability to trigger LLPS, and the small differences are also not in line with what could be verified in transfected HEK-Flp cells. Therefore, it is still hasty to use this type of algorithm as a tool when choosing and generating variants of interest with a limited number of changes.

### 3.2. Analysis of Phosphorylation/Dephosphorylation Dynamics of eIF2α in FT293-TIA1^WT/WDM^ Cell Lines under Oxidative Stress Conditions

**Figure 4:**
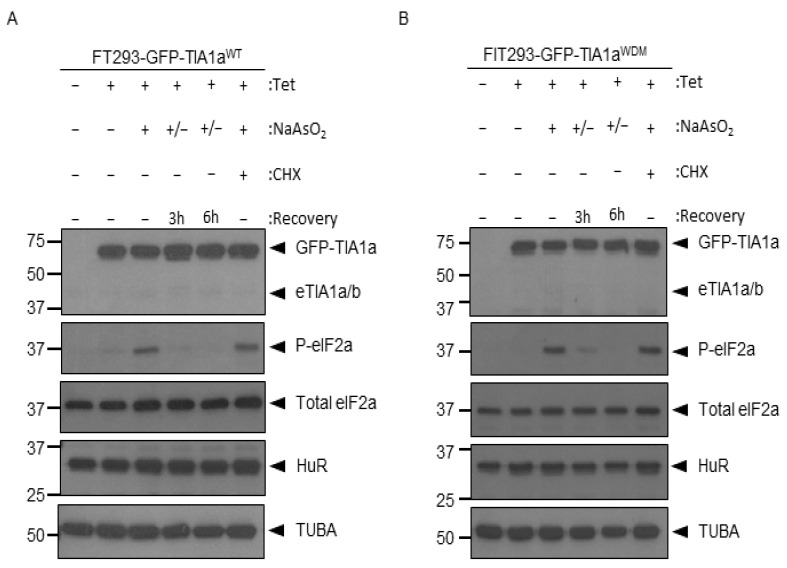
TIA1a^WT/WDM^ expression does not alter phosphorylation/dephosphorylation dynamics in an oxidative stress model. (**A**,**B**) Western blot of total cell extracts obtained from FT293 GFP-TIA1a^WT^ (**A**) and FT293 GFP-TIA1a^WDM^ (**B**) lines subjected to different treatments indicated in the legend at the top. Analyses were performed with anti-TIA1, anti-eIF2α-phosphorylated (P), anti-eIF2α (Total), anti-HuR, and anti-tubulin-α (TUBA) antibodies from three independent experiments. Molecular weight markers (kDa) and identification of each band are shown. The + and − signs indicate whether the sample contains the reagent (Tet, NaAsO_2_, CHX) or not. The +/− sign indicates that NaAsO_2_ is added and removed after 1 h. Abbreviations: Tet, tetracy-cline; NaAsO_2_, sodium arsenite; CHX, cycloheximide.

Phosphorylation of eIF2α is a hallmark feature of the inhibition of canonical 5′-cap-dependent translation, polysome disassembly, and SG formation that accompanies cellular stress [5,14,15,16,31,43]. Therefore, as a step prior to attempting to visualize the process of formation and disassembly of TIA1a^WT^- and TIA1a^WDM^-dependent SGs, and to establish the degree of stress from each of the cell lineages where we intended to visualize these processes, we analyzed the levels of phosphorylated (P) and total eIF2α in cell extracts of the stable FT293 GFP-TIA1a^WT^ and FT293 GFP-TIA1a^WDM^ lines. We quantified them after 1 h of treatment with sodium arsenite and at 3 and 6 h after removal of the stressful stimulus (Figure 4A,B). We also added an additional experimental condition, treatment with sodium arsenite and cycloheximide (CHX), an inhibitor of polysome disassembly, as a control that does not affect eIF2α phosphorylation (Figure 4A,B). In addition, the expression of other proteins, such as GFP-TIA1^WT^ and GFP-TIA1^WDM^ fusion proteins themselves; and other RNA-binding proteins (RBPs) such as HuR (Human antigen R); and TUBA, which was used as a loading control, were also analyzed (Figure 4A,B).

The results showed, firstly, that GFP-TIA1^WT^ or GFP-TIA1^WDM^ expression was null when no tetracycline was added, then the induction system is clean and lossless, and remained constant with the other indicated treatments. The endogenous expression of another RBP, such as HuR was also constant, demonstrating that their expression is not affected by oxidative stress induction (Figure 4A,B). In both the FT293 GFP-TIA1a^WT^ and FT293 GFP-TIA1a^WDM^ lines, 1 h of arsenite treatment resulted in a sharp increase in eIF2α phosphorylation, which decreased over subsequent hours of recovery, so that by 3 h only a small fraction of the factor remained phosphorylated, and by 6 h eIF2α-P was completely undetectable. Treatment with arsenite and cycloheximide (CHX) yielded the expected result, since, although this compound prevents the formation of SGs by inhibiting polysome disassembly, it does not interfere with eIF2α phosphorylation [14,17,43]. The levels of eIF2α-P in this case are equal to those achieved by arsenite-only treatment in both FT293 GFP-TIA1a^WT^ and GFP-TIA1a^WDM^. The expression of total eIF2α remained constant in all cases, so the changes seen in its phosphorylation are not due to changes in total protein levels, but to its modification. It is worth noting that FT293 GFP-TIA1a^WT^ cells show some phosphorylation of eIF2α, which is not observed in FT293 GFP-TIA1a^WDM^, even when no tetracycline is added or no arsenite stress is induced, so they might have been previously stressed by issues related to their culture.

Taken together, the results show that the phosphorylation/dephosphorylation dynamics of eIF2α are not affected by the mutation in TIA1^WDM^, but are similar between both variants. Therefore, it has been shown that 1 h of induction with arsenite is sufficient—both in cells expressing TIA1^WT^ and TIA1^WDM^—to be able to observe maximal SG formation without affecting cell viability. In turn, 3 h of arsenite induction is sufficient time to observe the process of SG disintegration, since after this time eIF2α returns to a state of dephosphorylation close to the basal state, suggesting that SGs will have almost completely disappeared.

### 3.3. Chronology of the Dynamics of Stress Granules in FT293 GFP-TIA1a^WT^ and GFP-TIA1a^WDM^ Cell Lines under Oxidative Stress

**Figure 5:**
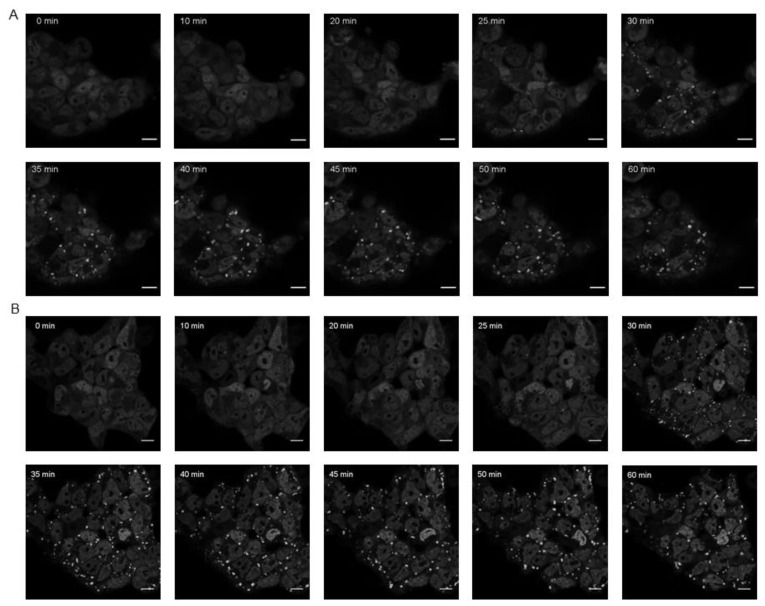
Timing of TIA1-dependent SG assembly in FT293-GFP-TIA1a^WT^ and FT293-GFP-TIA1a^WDM^ under oxidative stress. In vivo fluorescence microscopy images of FT293-GFP-TIA1a^WT^ (**A**) and FT293-GFP-TIA1a^WDM^ (**B**) lines expressing fusion proteins to GFP (grayscale). For each cell line, the formation of SGs for 1 h after addition of sodium arsenite is shown. The times at which the images have been taken since the addition of arsenite are shown at the top of each image. The scale bar corresponds to 10 μm.

**Figure 6:**
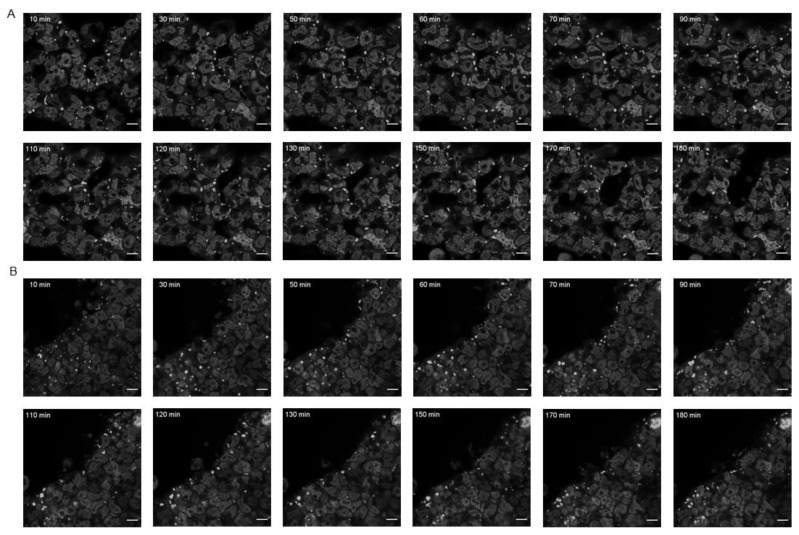
Timing of TIA1-dependent SG disassembly in FT293-GFP-TIA1a^WT^ and -GFP-TIA1a^WDM^ after oxidative stress. In vivo fluorescence microscopy images of FT293-GFP-TIA1a^WT^ (**A**) and FT293-GFP-TIA1a^WDM^ (**B**) lines expressing fusion proteins to GFP (grayscale). For each line, the disaggregation of SGs for 3 h after removal of sodium arsenite are shown. At the top of each image the times are indicated of when the images were taken after sodium arsenite removal. The indicated scale corresponds to 10 μm.

In vivo time-lapse fluorescence microscopy was used to monitor the formation and disaggregation of TIA1-dependent SGs in FT293-GFP-TIA1a^WT^ and FT293-GFP-TIA1a^WDM^ cell lines after induction of oxidative stress with sodium arsenite for 1 h (Figure 5 and Video S2) and subsequent withdrawal and recovery for 3 h (Figure 6 and Video S2). In FT293-GFP-TIA1a^WT^ cells, the first SGs started to be detected in the cytoplasm 25–30 min after inducing oxidative stress with arsenite under our experimental conditions, and it was observed how many of these SGs, initially of small size, coalesced during the ≃10–15 min thereafter to give rise to larger ones, which were maintained until the end of the observation. Thus, after 1 h, cells with SGs of heterogeneous sizes were observed (Figure 5A). However, nucleation of the aggregates would occur before 25 min, although they would still be too small and below the resolution limit of the microscope. In FT293-GFP-TIA1a^WDM^ cells, the dynamics of SG formation were similar, so that the first SGs started to be visible in the cytoplasm 30 min after the addition of the oxidizing agent (Figure 5B and Video S2). Again, it was possible to observe how SGs, initially small in size, coalesced to give rise to large SGs (Figure 5B). What is striking is the abundance of such large-sized aggregates in all cells at the end of the experiment, and the drastic decrease in the small-sized ones from which they originate, greater than that seen in FT293 GFP-TIA1a^WT^ cells (Figure 5B and Video S2). The images suggest that, in FT293 GFP-TIA1a^WDM^, SGs have higher fusion capacity so that they aggregate for longer, eventually forming larger SGs.

As for the recovery after arsenite removal, in FT293-GFP-TIA1a^WT^ cells the disintegration of SGs was greater during the first half hour, in which it was possible to see how the larger SGs subdivided into smaller ones until they disappeared. After that time, the decrease in the number and size of SGs was much more gradual, so that after 3 h after treatment removal, some small- and medium-sized SGs could still be detected in ≃25% of the cells (Figure 6A and Video S2). In FT293-GFP-TIA1a^WDM^ cells, the dynamics were a little different, and we could observe how many mainly small- and medium-sized SGs remained until 3 h of observation (Figure 6B and Video S2).

## 4. Discussion

**Figure 7:**
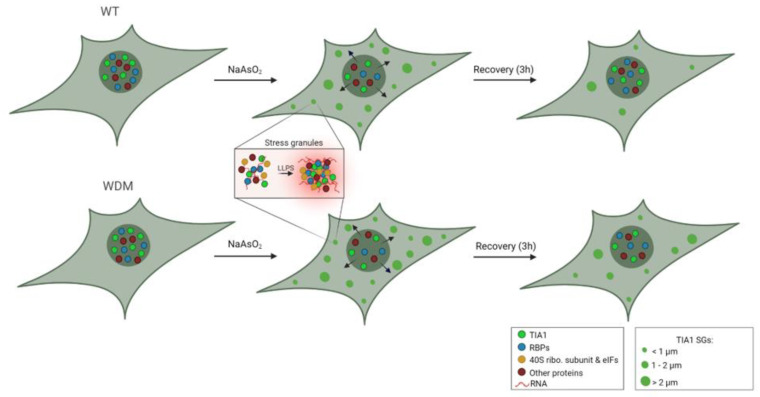
Dynamics of TIA1^WT/WDM^-dependent stress granules (SG) under oxidative stress by sodium arsenite. The acronyms WT and WDM represent the cell lines expressing either wild-type TIA1 (WT) or TIA1 containing the single mutation p.E384K associated with Welander distal myopathy (WDM). TIA1 and other RBPs are translocated from the nucleus to the cytoplasm, where they are nucleated; form SGs; and favor liquid–liquid phase separation (LLPS) together with other RBPs and non-RBPs, translation initiation factors, ribosomal subunits, coding, and non-coding RNAs; and inhibit the synthesis of non-essential proteins. This figure was created with BioRender.com.

In this study, we have attempted to clarify some molecular details that determine the formation and assembly/disassembly dynamics of TIA1-dependent SGs. To this aim, we have generated new variants of TIA1 that have allowed us to prove that, to alter SG dynamics—as observed in WDM—it is necessary for the amino acid that is modified with respect to TIA1^WT^ being replaced by a positively charged one. Furthermore, we have been able to confirm the chronology of SG formation (Figure 7).

TIA1 is one of the key components of SGs [14,15,16,26,31]. Previous studies have shown that the TIA1^WDM^ variant is associated with dysfunction in the formation of SGs, which are larger and more stable than those generated by TIA1^WT^ [25]. Dynamism is one of the most important properties of SGs, since it allows both their formation when facing a stressful situation, and their disassembly once homeostasis is recovered. The formation of SGs that are too stable can alter cellular homeostasis by affecting RNA metabolism (half-life or translation efficiency), cell signaling pathways, nucleo-cytoplasmic protein transport and/or RNAs, and direct or indirect sequestration of other cellular components whose abnormal distribution causes their malfunction and may eventually trigger cell death [33,36].

Recent studies have focused on analyzing the properties of the amino acids present in the PLD domains of some proteins, especially RBPs, as possible triggers for LLPS that eventually leads to the formation of SGs [28,29,42]. In this way, it has been shown that the interactions between certain amino acids of these PLDs and others present in RRMs are essential for the process to take place [28]. However, in TIA1, this disorganized C-terminal PLD domain does not adopt a single conformation that can be determined from the protein crystals that have been generated. Therefore, it is not possible to know for sure how it interacts with RRMs, if it does [28,29]. Regarding how C-terminal domains interact with each other when triggering LLPS, so far only some putative models have been developed [29]. These considerations have made it difficult to understand the role that E384K mutation can play in altering the intra- and intermolecular interactions of the protein.

Other in silico models, such as algorithms based on machine learning, have been developed in order to predict which proteins are more likely to trigger LLPS. The most up-to-date and reliable so far is the one developed by van Mierlo et al. (2021) [42]. However, its application to the sequence of TIA^WT^ and TIA^WDM^ yielded results that do not conform to those that have been contrasted in vivo, since it gave them a very low probability of triggering LLPS, when it is known that TIA1 is one of the main proteins involved in the formation of SGs and is capable of giving rise to LLPS even in vitro, in the presence of RNA or DNA [30,31,32]. Furthermore, the probability value obtained barely varied between TIA^WT^ and TIA^WDM^, nor did it when applying the algorithm on the variants generated in this work, when we have illustrated that there are in vivo differences in their ability to form SGs. This confirms that the in silico models are not yet sufficiently refined to help in the choice of the variants to generate in order to understand the effect of the punctual TIA1^WDM^ mutation, nor to replace the in vivo models. However, its refinement could make them a valuable tool in the future.

Therefore, given the need to continue using in vivo models to study the functions and alterations of TIA1, we designed variants of the protein trying to enhance the possible effects that the alteration of the charge of C-terminal residues of the protein could have [28]. The transfection of HEK cells with these variants, as well as TIA1a^WT^ and TIA1a^WDM^, allowed us to determine their effect on the formation of SGs. It was found that the mere overexpression of TIA1 in any of its variants leads to the spontaneous formation of SGs, which is consistent with previous observations [14,15,16,25,31]. However, this formation of SGs was much more pronounced with TIA1a^WDM^ and TIA1a^KKK^, so it suggests that substitution by one or three positive charges per se favors the ability of the protein to interact and aggregate [25]. On the other hand, cells transfected with TIA1a^WT^ generated fewer and smaller SGs when subjected to stress than those transfected with TIA1a^WDM^ or TIA1a^KKK^, as expected from previous tests carried out with TIA1a^WDM^ and TIA1^E384R^ [23,24,25]. However, it is striking that there are no significant differences in the amount or size of SGs generated between both variants, TIA1a^WDM^ and TIA1a^KKK^, since this suggests that the presence of a positive charge in this region, and not its intensity, is what alters the dynamics of SGs. Similarly, the SGs generated by these two variants were more persistent over time than those generated by TIA1a^WT^ or the other two variants, confirming that the presence of positive charges favors the interaction and stable aggregation of the protein [25].

For its part, TIA1a^AAA^ showed the same behavior as TIA1a^WT^ in terms of amount, size or stability of SGs, which suggests that the elimination of the negative charge provided by glutamic acid is not enough to alter the behavior of the protein and the formation of SGs. It must be replaced by a positive charge for this to happen. Finally, TIA1a^EEE^ also showed a similar behavior to TIA1a^WT^, except for the fact that the number of SGs it gave rise to after the addition of arsenite was slight but significantly lower, indicating that the increase in negative charges could make the interaction of the protein to form condensates difficult to some extent. Overall, the results agree with those obtained by Carrascoso et al. (2019) [25], who generated TIA1 variants mutating only the amino acid that changes WDM to others with different charges (arginine (positive), aspartic acid (negative), and glycine (no charge)). They were also able to verify that the positive charge favors the formation of SGs to a greater extent than the neutral charge, and this, in turn, was greater than the negative charge. Interestingly, they also determined that lysine in particular favors its formation over other positively charged amino acids, such as arginine, while they found no differences between negatively charged amino acids in maintaining the usual dynamics of SGs.

Furthermore, we also wanted to study processes that can only be analyzed in stable lines, for which we turned to the FT293 GFP-TIA1a^WT^ and GFP-TIA1a^WDM^ lines. The disassembly of polysomes that occurs after eIF2α phosphorylation is a necessary step for the formation of SGs in some cell types and/or stressors. Similarly, when phosphorylation is reversed, polysomes can reassemble and the canonical 5’-cap-dependent translation is re-established [5,14,15,16,31,43]. Our results have shown that the temporal pattern of phosphorylation/dephosphorylation of eIF2α is similar in cells expressing TIA1a^WT^ and TIA1a^WDM^: the levels of eIF2α-P that are reached after 1 h of induction of exogenous stress with arsenite are the same in both lines and they revert almost completely after 3 h of recovery, becoming eIF2α-P undetectable after 6 h. This corresponds to what was observed in this study, both in transient transfections and in vivo analyses, and to previous studies. We have been able to see how 30 min after treating the cells with arsenite, a massive formation of SGs is triggered so that, after 1 h, the maximum amount and size of SGs are reached, both in the transiently transfected lines and in the stable ones. It is also consistent with the analysis carried out by Carrascoso et al. (2019) [25], where the amount of SGs formed at 30 and 60 min after the application of stress was analyzed in lines expressing TIA1a^WT^ or TIA1a^WDM^, seeing a significant increase in the amount and size of SGs between these two points. For their part, Farny et al. (2009) proved in *Drosophila* lines that the increase in eIF2α-P levels between 1 and 2 h after the induction of stress is hardly noticeable, so we can conclude that 1 h of treatment is sufficient to observe the effects of stress induction on the formation of SGs [43].

Regarding the dephosphorylation of eIF2α when the stressful stimulus is eliminated, the fact that it occurs at the same rate in cells expressing TIA1a^WT^ or TIA1a^WDM^ indicates that it is not the cause of the delay in the disassembly of SGs that is observed when expressing TIA1a^WDM^. Recovery of homeostasis triggers the action of the phosphatase complex that acts on eIF2α [44], but the increase in intermolecular interactions between proteins and RNA found in the aggregates might be hindering the disassembly of the SGs independently. Similarly, phosphorylation of eIF2α is necessary, but not sufficient, for SGs to form, as demonstrated by applying compounds such as cycloheximide [15,17,43]. Therefore, phosphorylation of eIF2α and formation of SGs are processes which are related within the normal functioning of cells, but that can be unlinked when applying drugs or when there are mutated proteins, as in the case of TIA1^WDM^. However, we cannot rule out the existence of small differences in the timing of eIF2α dephosphorylation that could not be detected using this approach.

In vivo fluorescence microscopy is postulated as a very useful tool to obtain information on dynamic processes such as LLPS, since it allows us to see, not only the final result—as it would be in this case the number or size of the SGs—but also their evolution: formation, variation of the size and shape of the condensates over time, the flows they follow when merging or disintegrating, changes in their location, etc. [45]. This experimental approach has allowed us to establish and confirm the proposed timing for the formation of TIA1-dependent SGs, both in cells that express TIA1a^WT^ and TIA1a^WDM^. Regarding the formation after the addition of arsenite, we have been able to observe how SGs begin to be detectable after 30 min; first as small aggregates that fuse to give rise to larger ones. In cells that express TIA1a^WDM^, the proportion of larger SGs ends up being apparently higher in all cells, and it is intuited that the SG fusion process continues until reaching larger sizes, which is consistent with what has been previously described [23,24,25]. Regarding disassembly, we appreciate little differences between the cells expressing TIA1a^WT^ and TIA1a^WDM^, since it was observed how some small- and medium-sized SGs were maintained up to 3 h of observation (Figure 7).

## 5. Conclusions

This work has allowed us to gain further insight into the effects of the TIA1^WDM^ mutation on cell homeostasis and, particularly, on SG dynamics. Our result shows that the charge provided by the amino acids at the C-terminal end of the protein influences its ability to interact and aggregate and, thus, leads to abnormal and pathological SGs. Specifically, we have recapitulated and expanded the molecular and cellular details of the effects triggered by the substitution by a single positively charged amino acid on the dynamics of TIA1-dependent SGs. In the future, it will be interesting to continue generating variants that allow us to understand the characteristics of the C-terminal domain of the protein and to unravel how its ‘molecular grammar’ affects TIA1-mediated LLPS.

## Data Availability

Not applicable.

## Supplementary Materials

The following supporting information can be downloaded at: https://www.mdpi.com/article/10.3390/cells11050884/s1, Video S1: 3D-reconstitution of TIA1-dependent stress granules and its variants; Video S2: Videos of time lapse of TIA1-dependent SG assembly and disassembly in FT293-GFP-TIA1a^WT^ and FT293-GFP-TIA1a^WDM^ cell lines under oxidative stress; Video S3: Collection of the original images of Western blot analysis used to build the corresponding results shown in Figure 1 and Figure 4. Video S4: Disassembly SG GFP-TIA1a_WDM.
